# An assessment of PET and CMR radiomic features for the detection of cardiac sarcoidosis

**DOI:** 10.3389/fnume.2024.1324698

**Published:** 2024-01-16

**Authors:** Nouf A. Mushari, Georgios Soultanidis, Lisa Duff, Maria G. Trivieri, Zahi A. Fayad, Philip Robson, Charalampos Tsoumpas

**Affiliations:** ^1^ Leeds Institute of Cardiovascular and Metabolic Medicine, University of Leeds, Leeds, United Kingdom; ^2^ BioMedical Engineering and Imaging Institute, Icahn School of Medicine at Mount Sinai, New York, NY, United States; ^3^ Beatson Institute for Cancer Research, University of Glasgow, Glasgow, United Kingdom; ^4^ Cardiovascular Research Institute, Icahn School of Medicine at Mount Sinai, New York, NY, United States; ^5^ Department of Nuclear Medicine and Molecular Imaging, University Medical Centre Groningen, University of Groningen, Groningen, Netherlands

**Keywords:** cardiac sarcoidosis, post-COVID, PET-MRI, imaging, machine learning

## Abstract

**Background:**

Visual interpretation of PET and CMR may fail to identify cardiac sarcoidosis (CS) with high specificity. This study aimed to evaluate the role of [^18^F]FDG PET and late gadolinium enhancement (LGE)-CMR radiomic features in differentiating CS from another cause of myocardial inflammation, in this case patients with cardiac-related clinical symptoms following COVID-19.

**Methods:**

[^18^F]FDG PET and LGE-CMR were treated separately in this work. There were 35 post-COVID-19 (PC) and 40 CS datasets. Regions of interest were delineated manually around the entire left ventricle for the PET and LGE-CMR datasets. Radiomic features were then extracted. The ability of individual features to correctly identify image data as CS or PC was tested to predict the clinical classification of CS vs. PC using Mann–Whitney *U*-tests and logistic regression. Features were retained if the *P*-value was <0.00053, the AUC was >0.5, and the accuracy was >0.7. After applying the correlation test, uncorrelated features were used as a signature (joint features) to train machine learning classifiers. For LGE-CMR analysis, to further improve the results, different classifiers were used for individual features besides logistic regression, and the results of individual features of each classifier were screened to create a signature that included all features that followed the previously mentioned criteria and used it them as input for machine learning classifiers.

**Results:**

The Mann–Whitney *U*-tests and logistic regression were trained on individual features to build a collection of features. For [^18^F]FDG PET analysis, the maximum target-to-background ratio (*TBR_max_*) showed a high area under the curve (AUC) and accuracy with small *P*-values (<0.00053), but the signature performed better (AUC 0.98 and accuracy 0.91). For LGE-CMR analysis, the *Gray Level Dependence Matrix (gldm)-Dependence Non-Uniformity* showed good results with small error bars (accuracy 0.75 and AUC 0.87). However, by applying a Support Vector Machine classifier to individual LGE-CMR features and creating a signature, a Random Forest classifier displayed better AUC and accuracy (0.91 and 0.84, respectively).

**Conclusion:**

Using radiomic features may prove useful in identifying individuals with CS. Some features showed promising results in differentiating between PC and CS. By automating the analysis, the patient management process can be accelerated and improved.

## 1 Introduction

Cardiac sarcoidosis (CS) is a granulomatous inflammatory disease that can be diagnosed with [^18^F]-fluorodeoxyglucose positron emission tomography ([^18^F]FDG PET). [^18^F]FDG PET is performed in suspected CS due to the avid uptake of glucose by the active inflammation cells in sarcoid granulomas. It is recommended that a low-carbohydrate, high-fat diet followed by fasting be used to inhibit the physiologic glucose metabolism of the heart to enable diagnostic imaging. Moreover, a cardiac PET with abnormal [^18^F]FDG uptake on suppressed myocardial uptake is crucial to CS diagnosis (1). A PET image can also be used to quantify inflammation in addition to a visual review. Several metrics exist to describe the intensity and heterogeneity of [^18^F]FDG uptake. PET is less specific for CS when there is no extracardiac uptake (2). In addition, it is critical to note that approximately 25% of cardiac PET studies fail due to the inadequate suppression of physiologic glucose uptake (3).

Conversely, cardiovascular magnetic resonance (CMR) is a non-invasive imaging technique that plays a significant role in diagnosing or screening patients with CS. It can detect scar tissue that may indicate inactive CS (4). Myocardial scarring can be evaluated using late gadolinium enhancement (LGE) imaging. Gadolinium is an extracellular contrast agent that exhibits a slow washout in fibrotic regions compared to the normal myocardium. Although LGE is helpful in identifying CS, based on the distribution and pattern of LGE (5, 6), it is a non-specific tool. In addition, LGE-CMR has limited sensitivity prior to the development of myocardial scar (7).

Moreover, [^18^F]FDG PET can detect the inflammation related to CS, which theoretically leads to its early diagnosis (8). On the other hand, CMR with LGE is capable of identifying myocardial scarring even in small areas, owing to its high spatial resolution. The specificity of CMR in diagnosing CS might be higher than [^18^F]FDG PET; however, both have high sensitivity (9). There is controversy among studies regarding the identification of the appropriate technique for diagnosing CS (10–13). Similarly, the feasibility of combining the findings of both [^18^F]FDG PET and LGE-CMR has not been adequately explored; this could enhance the accuracy of the assessment by identifying different pathologic features.

Additionally, it may be possible to gain additional information by employing quantitative measurements that may provide complementary information greater than that provided by non-invasive methods (14). A method of analyzing imaging data uses radiomics to automatically extract high-dimensional features. Subsequently, researchers can mine and analyze these features to support decision-making (15, 16). First-order statistical features comprise properties based on histograms (HISTO). Regardless of the spatial relationship between the voxels, these features are based on the shape of the histogram and statistical values of the voxel intensities (17, 18). Statistical inter-relationships between neighboring voxels are calculated using second-order statistical features, which can be derived from the gray-level cooccurrence matrix (GLCM) (17). In addition, areas with coarser textures can be extracted using higher-order statistical features (19). These are derived from the gray level run length matrix (GLRLM), the gray level dependence matrix (GLDM), the gray level size zone matrix (GLSZM), and the neighboring gray tone difference matrix (NGTDM).

Correspondingly, this work investigates the precision of PET and CMR radiomic features in differentiating CS from another cause of myocardial inflammation, in this case, patients with cardiac-related symptoms following COVID-19, or post-COVID-19 (PC) patients. Myocardial inflammation can be a symptom observed in some PC patients. The severity and prevalence of myocardial inflammation may vary among individuals, and it is one of the potential complications associated with COVID-19. It is important to note that not all PC patients will experience myocardial inflammation, and the manifestation of symptoms can vary widely (20).

## 2 Materials and methods

### 2.1 Ethical approval

This study was conducted with the approval of the Institutional Review Board at Mount Sinai Hospital (GCO # 01-1032). All the subjects supplied their written, informed consent.

### 2.2 Subject selection

**Figure 1:**
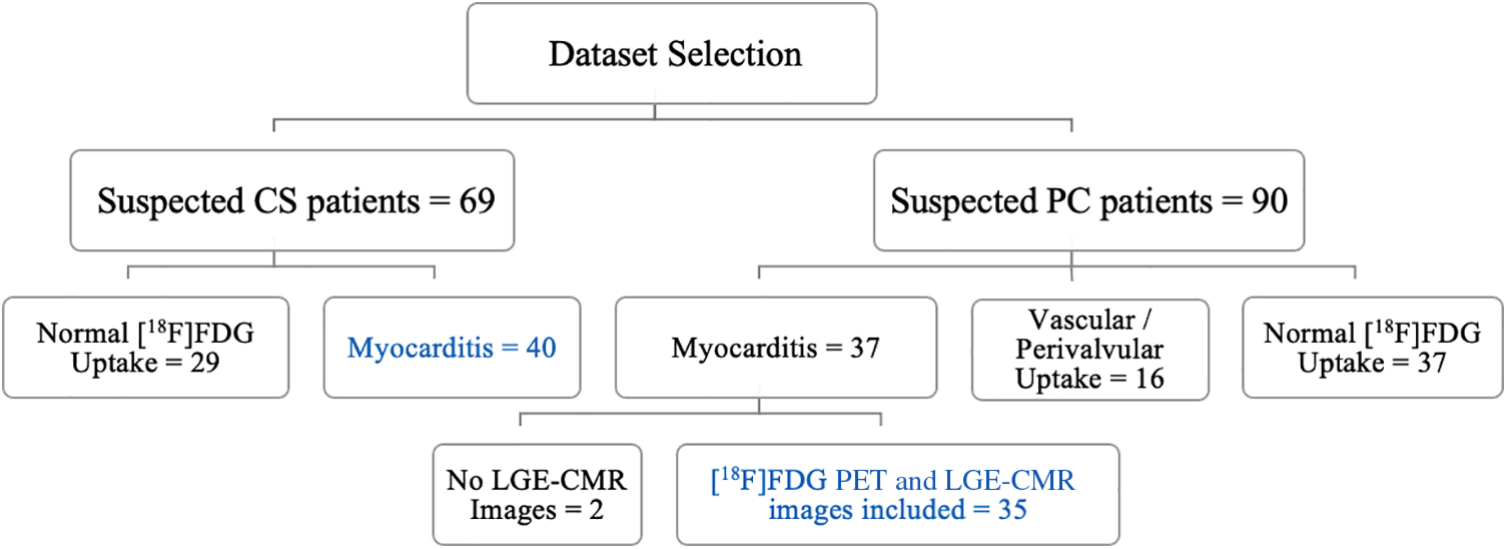
A flowchart of dataset selection.

Both PET and CMR imaging were performed at Mount Sinai Hospital in New York on two types of patients: patients suspected of having cardiac sarcoidosis due to extracardiac disease and cardiac symptoms, and PC patients. The majority of the CS cohort predates the COVID-19 era, ensuring that these patients did not exhibit post-COVID-19 symptoms. The CS diagnosis is consistent with the Heart Rhythm Society (HRS) expert consensus statement (10). PC patients had either chest pain, palpitations, or shortness of breath following COVID-19 that could not be attributed to another cause. This retrospective study encompassed CS and PC patients exhibiting abnormal FDG uptake in the myocardium who were evaluated by a cardiologist who is an expert in the use of PET/MR for the diagnosis of cardiomyopathies. Exclusions were made for individuals with renal dysfunction, insulin-dependent diabetes, blood glucose levels exceeding 200 mg/dl, pregnant or lactating individuals, and those with cardiac pacemakers or automatic implantable cardioverter-defibrillators. In preparation for the scan, the patient was required to abstain from carbohydrate consumption for 24 h and fast for 12 h. Initially, there were 90 suspected PC patients and 69 patients with CS. However, for the purpose of this study, only cases with myocarditis were included. Therefore, the study included 35 datasets from PC patients and 40 datasets from patients with CS, as summarized in Figure 1. The demographic information of the patients is provided in Table 1.

**Table 1:** Demographic information of the study population.

Group	Sex	Mean age	Standard deviation
PC	F = 19	44.2	12.27
	M = 16		
CS	F = 16	61.35	9.41
	M = 24		

PC, post-COVID-19 patients; CS, cardiac sarcoidosis patients.

### 2.3 Imaging protocol

An integrated PET/MR system was used to perform simultaneous CMR and [^18^F]FDG PET (Biograph™ mMR, Siemens Healthcare, Erlangen, Germany). An intravenous injection of [^18^F]FDG containing 5 MBq/kg was given to the patients. Acquisition of thoracic PET (one-bed position centered on the heart) takes approximately 90 min to scan the patients in two phases (blood and tissue phases). However, for the purpose of this study, only the last 60 min of the time window were chosen because the focus of this study specifically centers on the tissue phase. Iterative ordinary Poisson ordered subset expectation maximization (OP-OSEM) was used to reconstruct PET images over a 344 × 344 × 129 image matrix with 3 iterations, 21 subsets, and 2 mm isotropic voxels, followed by post-filtering using a Gaussian kernel of 4 mm. The PET study was neither respiratory-gated nor electrocardiogram (ECG)-gated, and no motion correction was carried out. Attenuation correction was performed using a 3D breath-hold Dixon-based MR image. Parallel to the PET scan, CMR was performed with ECG triggering covering the whole left ventricle. Inversion-recovery gradient-echo LGE sequences were acquired across the entire myocardium approximately 15 min after injection of a 0.2 mmol/kg gadolinium-based contrast agent (MultiHance, Bracco, NJ) with 8 mm slice thickness and 10 mm spacing between slices. Bias correction was not performed on CMR images.

### 2.4 Segmentation

**Figure 2:**
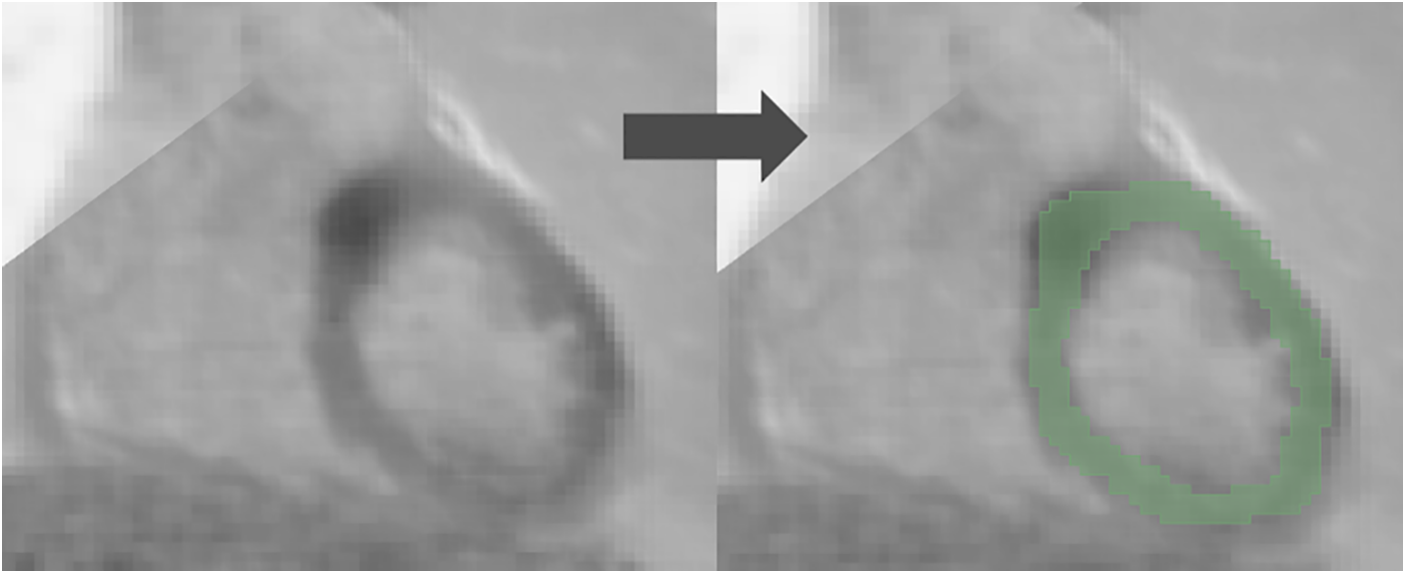
An example of the segmentation on a PET/CMR image.

3D slicer software (Version 4.11.2; https://www.slicer.org) was used for the segmentation (21, 22). Regions of interest (ROI) were drawn manually in the entire left ventricular myocardium for both [^18^F]FDG PET and LGE-CMR images by a junior radiographer and reviewed by a Biomedical Engineering expert with 10 years of experience in Medical Imaging. This approach is less likely to be influenced by the intensity and experience of observers compared to the hot regions-only segmentation. The hot regions-only segmentation may exhibit bias and result in unreliable outcomes during testing in our prior study (23). Figure 2 provides an illustrative example of the segmentation on the PET/CMR images. Subsequently, radiomic features were extracted.

To calculate the maximum target-to-background ratio (TBR_max_) in PET images, the standardized uptake value (SUV_max_) was extracted, and another ROI was drawn in the blood pool to extract the (SUV_mean_) of the background and then follow the following equation (1):

(1) $$\mathrm{TB}R_{\max}=\frac{\mathrm{SU}V_{\max}\;(\mathrm{target})}{\mathrm{SU}V_{\mathrm{mean}}\;(\mathrm{background})}$$

### 2.5 Feature extraction

PyRadiomics (Version 3.0.1) was used to extract six feature classes (totaling 94 features) from the PET/CMR images (24). A list of all radiomic features is shown in Supplementary Material S1. PyRadiomics adheres to most of the image biomarker standardization initiative's (IBSI) feature definitions. In the case of PET images, a fixed bin size of 0.075 was utilized, which gave a good number of bins and a good representation of the data. However, for LGE-CMR images, the default fixed bin size of 25 was used. The impact of gray-level discretization on extracted feature values from PET images has been well documented (25). Nevertheless, there is limited research exploring the effect of gray-level discretization on clinical MR images. According to Duron et al.'s (26) experimental study, aimed at examining the impact of gray-level discretization on the reproducibility of texture features from MR images, utilizing different fixed bin sizes had a minimal effect on the variability of these features. The PET images were subjected to SUV normalization. Since the datasets were obtained from a single scanner, pre-processing (except post-filtering using a Gaussian kernel in the reconstruction process in PET images) and harmonization were not performed. The feature extraction was conducted in 3D, as it provides more informative results compared to 2D analysis. In addition, to mitigate the risk of overfitting caused by limited data, the models were not optimized.

### 2.6 Statistical analysis

**Figure 3:**
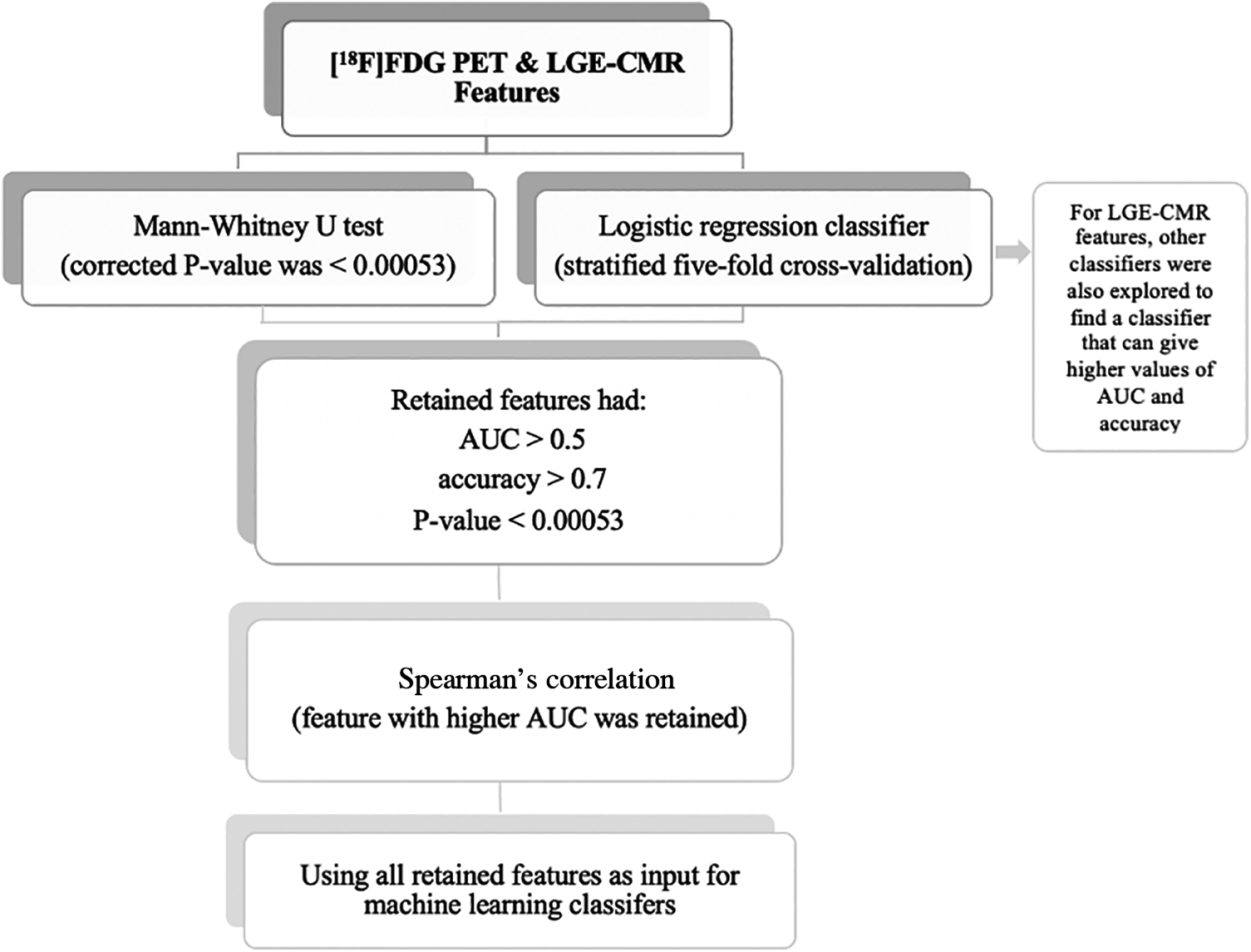
Statistical analysis workflow.

Statistical analyses were undertaken using the Scikit-learn software (Version 0.23.2) (27). The individual radiomic features of the study groups were compared using the Mann-Whitney U test to assess their ability to separate CS from PC. In addition, the Bonferroni correction was used to adjust the *P*-value for multiple tests. According to the significance level of 0.05, with 94 features, the corrected *P*-value was <0.00053. The radiomic features were then trained and tested using logistic regression classifiers. This analysis used stratified five-fold cross-validation to obtain the mean area under the curve (AUC), mean accuracy, and 95% confidence intervals (CIs). An AUC >0.5 and an accuracy >0.7 were considered acceptable for the retention of features with a *P*-value of less than 0.00053. When 0.5 < AUC < 1, there is a high chance that the feature will be able to distinguish the positive class values from the negative ones. In addition, accuracy >0.7 can be considered a decent score. Subsequently, Spearman correlation was used to detect the correlated features with a 0.70 correlation coefficient. This threshold was selected because higher thresholds indicate a strong similarity between the two features, with at least half of their variance being shared. Of these correlated features, the feature with the highest AUC was retained. Following that, the uncorrelated features were then used as input for the machine learning classifiers to create a signature (joint features). In LGE-CMR features, to find a classifier that can provide high values of AUC and accuracy, other classifiers besides logistic regression were explored. The retained features were then used as input for machine learning classifiers. The selection of the top-performing machine learning classifier was based on the highest mean AUC and mean accuracy values from stratified five-fold cross-validation. Due to the small sample size in this study, only the training cross-validation outcomes were documented. This approach has been recommended in situations where the sample size is insufficient to support an independent validation set (28). By using cross-validation, the potential overestimation of the model's performance was reduced. The workflow of the statistical analysis is illustrated in Figure 3. In this study, the PET and CMR datasets were analyzed separately, allowing for a more focused investigation of the specific features and characteristics inherent to each modality. This approach yields valuable insights into the individual contributions of PET and CMR, enhancing the understanding of the subject under investigation.

## 3 Results

### 3.1 Individual feature assessment

The univariate analysis of individual features in each dataset revealed that the [^18^F]FDG PET and LGE-CMR dataset had five and 11 features, respectively, with *P*-values <0.00053. For all datasets, Table 2 shows the five best radiomic features based on the *P*-values.

**Table 2:** Five best radiomic features based on *P*-values.

PET features	*P*-value	LGE-CMR features	*P*-value
*TBR_max_*	1.5 × 10^−11^	*glszm_Small Area Low Gray Level Emphasis*	7.2 × 10^−7^
*glszm_Large Area High Gray Level Emphasis*	2 × 10^−5^	*gldm_Dependence Non-Uniformity*	7.3 × 10^−7^
*glrlm_Gray Level Non-Uniformity*	1 × 10^−4^	*gldm_Small Dependence Low Gray Level Emphasis*	8.8 × 10^−7^
*gldm_Gray Level Non-Uniformity*	1.2 × 10^−4^	*glszm_Low Gray Level Zone Emphasis*	1.3 × 10^−6^
*glszm_Zone Variance*	1.8 × 10^−4^	*glrlm_Run Length Non-Uniformity*	8.8 × 10^−6^

GLSZM, gray level size zone matrix; GLRLM, gray level run length matrix; GLDM, gray level dependence matrix.

### 3.2 Feature selection

The logistic regression was applied to individual features. [^18^F] The PET and LGE-CMR datasets had only three and five features, respectively, conforming to the inclusion criteria (*P*-value <0.00053, AUC >0.5, and accuracy >0.7). Those features that met the inclusion criteria were again screened based on correlation. To detect the correlated features, a correlation test was conducted. Features with a higher AUC were retained. The number of selected features in the PET and LGE-CMR features decreased to two uncorrelated features for each one. Table 3 presents the AUC and accuracy values, along with their corresponding 95% CI, for the uncorrelated features in each dataset. Scrutinizing PET features in greater detail, *TBR_max_* conveyed high AUC and accuracy with relatively small confidence intervals while *glszm_Zone Variance* had acceptable values but large confidence intervals. Creating a signature using these uncorrelated features as input for machine learning classifiers improved the performance. Random Forest was the best one (95% CI AUC 0.95–1.00: accuracy 0.83–0.99). The performance of all machine learning classifiers is displayed in Table 4.

**Table 3:** Areas under the curve (AUCs) and accuracies (ACC) of uncorrelated features.

	Feature	ACC	ACC CI	AUC	AUC CI	Sensitivity	Specificity
PET	*TBR_max_*	0.89	0.07	0.95	0.09	0.91	0.88
	*glszm_zone variance*	0.71	0.15	0.69	0.22	0.49	0.90
LGE-CMR	*gldm_dependence non-uniformity*	0.75	0.06	0.87	0.05	0.69	0.80
	*glrlm_long run high gray level emphasis*	0.71	0.15	0.78	0.21	0.57	0.83

CI, confidence interval; GLSZM, gray level size zone matrix; GLDM, gray level dependence matrix; GLRLM, gray level run length matrix.

**Table 4:** Machine learning classifier performance of PET joint features with 95% confidence intervals (CI).

Machine learning classifier	ACC	ACC CI	AUC	AUC CI	Sensitivity	Specificity
Random forest	0.91	0.08	0.98	0.03	0.94	0.90
Logistic regression	0.87	0.09	0.96	0.07	0.83	0.90
Support vector machine	0.63	0.08	0.56	0.31	0.26	0.95
Decision tree	0.88	0.03	0.88	0.04	0.86	0.93
Gaussian process	0.61	0.10	0.70	0.16	0.40	0.80
Stochastic gradient descent	0.48	0.03	0.71	0.19	0.80	0.20
Perceptron	0.44	0.07	0.69	0.22	0.94	0.00
Passive aggressive	0.63	0.12	0.69	0.31	0.40	1.00
Neural network	0.53	0.17	0.64	0.12	0.97	0.20
K-neighbors	0.69	0.04	0.73	0.11	0.60	0.78

ACC, accuracy; AUC, area under the curve.

**Figure 4:**
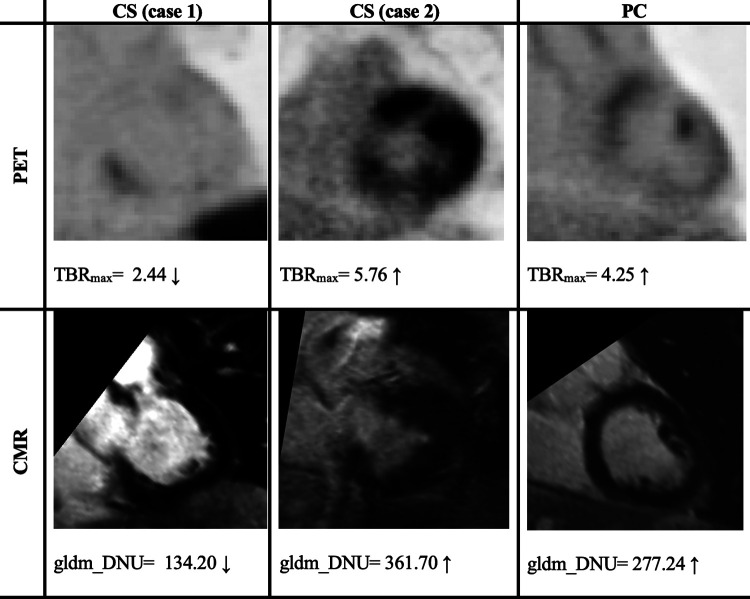
Two cases of PET/CMR cardiac sarcoidosis (CS) and one case of post-COVID-19 (PC) associated with the best-performance features. PET/CMR CS (case 1) has significantly lower values than PC values, whereas (case 2) has values in the range of PC patients’ values, potentially leading to a misdiagnosis. The display intensity of PET images ranges from 0 to 6.

For LGE-CMR features, *gldm_Dependence Non-Uniformity* presented good AUC and accuracy (95% CI AUC 0.82–0.92: accuracy 0.69–0.81). Examples of PET and CMR images for CS and PC with related features are shown in Figure 4. However, to further improve the AUC and accuracy findings of LGE-CMR, additional measures were taken. After applying many other classifiers besides logistic regression, the Support Vector Machine showed six features following the criteria (Table 5). The total number of features was not normalized before applying the Support Vector Machine because the focus was on the relative relationships between the data points rather than their absolute values or scales. Additionally, normalization had the potential to modify the original distances and influence the underlying relationships within the data, which are crucial for distance-based algorithms. The retained features were used as input for machine learning classifiers, and Random Forest proved to have the greatest AUC and accuracy values (95% CI AUC 0.82–1.00: accuracy 0.73–0.95). The machine learning classifiers performance for joint features is shown in Table 6.

**Table 5:** Areas under the curve (AUCs) and accuracies (ACC) of uncorrelated features using the support vector machine (SVM) that used to create a CMR signature.

	Feature	ACC	ACC CI	AUC	AUC CI	Sensitivity	Specificity
SVM classifier	*glszm_Low Gray Level Zone Emphasis*	0.72	0.18	0.83	0.17	0.66	0.78
	*glrlm_Run Entropy*	0.72	0.14	0.80	0.14	0.77	0.68
	*glszm_Small Area Low Gray Level Emphasis*	0.81	0.12	0.79	0.17	0.63	0.98
	*gldm_Dependence Non-Uniformity*	0.73	0.09	0.78	0.11	0.69	0.78
	*gldm_Small Dependence Low Gray Level Emphasis*	0.76	0.12	0.77	0.17	0.60	0.90
	*glrlm_Gray Level Non-Uniformity*	0.71	0.11	0.66	0.16	0.46	0.93

CI, confidence interval; GLSZM, gray level size zone matrix; GLRLM, gray level run length matrix; GLDM, gray level dependence matrix.

**Table 6:** Machine learning classifier performance of LGE-CMR joint features with 95% confidence intervals (CI).

Machine learning classifier	ACC	ACC CI	AUC	AUC CI	Sensitivity	Specificity
Random forest	0.84	0.11	0.91	0.09	0.77	0.93
Logistic regression	0.77	0.07	0.88	0.07	0.74	0.80
Support vector machine	0.72	0.15	0.79	0.15	0.51	0.90
Decision tree	0.75	0.11	0.75	0.11	0.74	0.75
Gaussian process	0.53	0.09	0.52	0.04	0.20	0.83
Stochastic gradient descent	0.48	0.03	0.62	0.09	0.49	0.53
Perceptron	0.55	0.03	0.24	0.15	0.03	1.00
Passive aggressive	0.49	0.04	0.58	0.34	0.54	0.45
Neural network	0.68	0.19	0.78	0.26	0.71	0.33
K-neighbors	0.68	0.08	0.69	0.09	0.60	0.75

ACC, accuracy; AUC, area under the curve.

## 4 Discussion

CS is an inflammatory disease with an unknown cause. To aid in the diagnostic process, advanced imaging techniques like [^18^F]FDG PET and LGE-CMR are recommended. [^18^F]FDG PET is utilized in suspected CS cases due to its ability to detect glucose uptake by active inflammatory cells in sarcoid granulomas, while LGE-CMR can identify scar tissue that may indicate inactive CS. However, both techniques have limitations that contribute to their lack of specificity for CS. This study focused on investigating the potential of [^18^F]FDG PET and LGE-CMR radiomic features in differentiating CS from other causes of myocardial inflammation, specifically in patients with post-COVID-19 symptoms related to the heart.

After applying several steps to filter the radiomic features of PET images, *TBR_max_* succeeded in being the best-performing feature. *TBR_max_* was able to discriminate approximately 90% of CS cases from PC cases. The majority of the CS cases had a *TBR_max_* range between 1 and 3, while PC cases had higher values. This result is supported by other studies that revealed similar range values of *TBR_max_* in CS patients, which were between 1 and 3 (12, 23). To some extent, *TBR_max_* can make fair comparisons between institutions by looking at the equation for extracting their values, which essentially means a blood uptake correction (29). Although *TBR_max_* has successfully discriminated approximately 90% of cases, there are still approximately 10% of cases that have been misdiagnosed, such as the *TBR_max_* value of case 2 in Figure 4, which provided values that were approximately similar to those of PC patients. *glszm_Zone Variance* was the second-best-performing feature but had significant error bars that made it unreliable. However, the PET feature performance improved significantly after using joint features as input for machine learning classifiers, especially the Random Forest classifier.

For the radiomic features of CMR images, *gldm_Dependence Non-Uniformity* presented acceptable results, but with some errors. Approximately 67.5% of CS patients had values less than 88.5% of PC patients (average value in CS = 205.8, average value in PC = 323.4). This measure determines the degree of similarity of dependence within an ROI (24). Therefore, the higher values of *gldm_Dependence Non-Uniformity* indicate a greater level of heterogeneity. As an interpretation of the values of each group, CS appears to have a lower variance than PC. However, it is recommended that this feature be interpreted cautiously because it may contain errors, especially for the CS group, as one-third of CS patients had values similar to those of the majority of PC patients. In Figure 4, it can be seen that there was a big difference between the feature values in the first and second cases, and the second case even gave higher values than the PC patient value.

One of the approaches followed to augment the performance of the LGE-CMR features is creating a signature (joint features) that includes all the uncorrelated features with the best AUCs and good accuracies. This step was applied to the output of the logistic regression as well as other classifiers. The signature from the Support Vector Machine illustrated great results and ameliorated the findings compared to the individual features. The individual feature of the LGE-CMR dataset from logistic regression, *gldm_Dependence Non-Uniformity*, had a lower AUC but smaller confidence intervals than when using the signature. Employing the joint features gives only a little advantage in making it the superior choice for LGE-CMR dataset classification.

In our previous study (23), *gldm_Dependence Non-Uniformity* emerged as one of the top features in PET images for distinguishing between CS patients and controls. However, it exhibited larger error bars compared to *TBR_max_*, indicating greater variability in its measurements. In contrast, the evaluation of LGE-CMR radiomic features to differentiate between active CS and inactive CS (4) yielded different top features compared to the current study. This discrepancy can be attributed to the distinct types of comparisons conducted in each study and to slight variations in the methodology employed. These alterations resulted in improved outcomes from the analysis of LGE-CMR images. Radiomic analysis is affected by several factors that make comparisons between studies difficult. Findings across studies are not consistently replicated; instead, they often exhibit conflicting results (30, 31). This divergence in outcomes could potentially be attributed to technical factors. Efforts should be made to minimize variation up to the reconstruction step to ensure consistency. It is crucial to avoid introducing variation in factors that occur after reconstruction whenever possible. This entails making consistent choices, such as employing the same image segmentation algorithm and utilizing a uniform discretization scheme for all the data (32). In addition, the higher the resolution and number of voxels, the more they can impact certain radiomic features by inflating their values (33). By mitigating variability at these stages, the reliability and comparability of the results can be enhanced.

This study has several limitations. First, all studies evaluating patients with suspected CS have well-known limitations due to the lack of a standard for diagnosing the condition. However, it is possible to detect CS more effectively by combining data from both CMR and PET. Endomyocardial biopsies were not routinely performed in this cohort of patients. It is, however, difficult to rule out CS with an endomyocardial biopsy due to its low sensitivity and high sampling error rate because of its focal distribution (34). In addition, considering the sample size, further studies are needed to verify this conclusion to avoid overfitting and type I errors. This issue was reduced by applying the Bonferroni correction. Furthermore, validating the AI approach on a larger and more diverse patient population, as well as normal controls, would indeed increase the robustness and applicability of the results. Moreover, no automated segmentation was performed, and reference segmentation was not provided in this study.

The novel finding of this study is that radiomic analysis can enhance the objectivity and complementarity of PET and CMR in identifying CS from PC. PET-based analysis could effectively differentiate CS from PC. The PET joint features demonstrated high performance, which can be used alone without resorting to CMR. However, CMR-based analysis is helpful when PET images suffer from failed suppression of the physiological uptake of [^18^F]FDG in the myocardium (3). Results may vary from one institution to another due to different scanning procedures and protocols, and to the characteristics of each scanner. However, the methodology is straightforward and transferable to PET/CT-only and MRI-only studies.

## 5 Conclusion

This work adds to the growing evidence that radiomic analysis may assist [^18^F]FDG PET and LGE-CMR in precisely discerning cardiac sarcoidosis, with a specific focus on *TBR_max_*. These features hold promise for heightening the accuracy of diagnoses. Nonetheless, more research is warranted to validate and refine these results and guarantee their wider clinical applicability.

## Data Availability

The original contributions presented in the study are included in the article/Supplementary Material, further inquiries can be directed to the corresponding author.
