# Supplementary material for: An assessment of PET and CMR radiomic features for the detection of cardiac sarcoidosis
Source: Front Nucl Med. 2024 Jan 16;4:1324698. doi: 10.3389/fnume.2024.1324698 (PMC11460291; doi:10.3389/fnume.2024.1324698)
Supplement: Supplementary file 1 [file Table1.docx]

Supplementary Material 1

An assessment of PET and CMR radiomic features for detection of cardiac sarcoidosis

Table 1 List of all radiomic features

| First order features | | |
| --- | --- | --- |
| firstorder_10Percentile | firstorder_Minimum | |
| firstorder_90Percentile | firstorder_Range | |
| firstorder_Energy | firstorder_RobustMeanAbsoluteDeviation | |
| firstorder_Entropy | firstorder_RootMeanSquared | |
| firstorder_InterquartileRange | firstorder_Skewness | |
| firstorder_Kurtosis | firstorder_TotalEnergy | |
| firstorder_Maximum | firstorder_Uniformity | |
| firstorder_MeanAbsoluteDeviation | firstorder_Variance | |
| firstorder_Mean | TBRmax (for PET only) | |
| firstorder_Median |  | |
| GLCM features | | |
| glcm_Autocorrelation | glcm_Idn | |
| glcm_ClusterProminence | glcm_Imc1 | |
| glcm_ClusterShade | glcm_Imc2 | |
| glcm_ClusterTendency | glcm_InverseVariance | |
| glcm_Contrast | glcm_JointAverage | |
| glcm_Correlation | glcm_JointEnergy | |
| glcm_DifferenceAverage | glcm_JointEntropy | |
| glcm_DifferenceEntropy | glcm_MCC | |
| glcm_DifferenceVariance | glcm_MaximumProbability | |
| glcm_Id | glcm_SumAverage | |
| glcm_Idm | glcm_SumEntropy | |
| glcm_Idmn | glcm_SumSquares | |
| GLRLM features | | |
| glrlm_GrayLevelNonUniformity | glrlm_RunEntropy | |
| glrlm_GrayLevelNonUniformityNormalized | glrlm_RunLengthNonUniformity | |
| glrlm_GrayLevelVariance | glrlm_RunLengthNonUniformityNormalized | |
| glrlm_HighGrayLevelRunEmphasis | glrlm_RunPercentage | |
| glrlm_LongRunEmphasis | glrlm_RunVariance | |
| glrlm_LongRunHighGrayLevelEmphasis | glrlm_ShortRunEmphasis | |
| glrlm_LongRunLowGrayLevelEmphasis | glrlm_ShortRunHighGrayLevelEmphasis | |
| glrlm_LowGrayLevelRunEmphasis | glrlm_ShortRunLowGrayLevelEmphasis | |
| GLSZM features | | |
| glszm_GrayLevelNonUniformity | glszm_SizeZoneNonUniformity | |
| glszm_GrayLevelNonUniformityNormalized | glszm_SizeZoneNonUniformityNormalized | |
| glszm_GrayLevelVariance | glszm_SmallAreaEmphasis | |
| glszm_HighGrayLevelZoneEmphasis | glszm_SmallAreaHighGrayLevelEmphasis | |
| glszm_LargeAreaEmphasis | glszm_SmallAreaLowGrayLevelEmphasis | |
| glszm_LargeAreaHighGrayLevelEmphasis | glszm_ZoneEntropy | |
| glszm_LargeAreaLowGrayLevelEmphasis | glszm_ZonePercentage | |
| glszm_LowGrayLevelZoneEmphasis | glszm_ZoneVariance | |
| GLDM features | | |
| gldm_DependenceEntropy | | gldm_LargeDependenceEmphasis |
| gldm_DependenceNonUniformity | | gldm_LargeDependenceHighGrayLevelEmphasis |
| gldm_DependenceNonUniformityNormalized | | gldm_LargeDependenceLowGrayLevelEmphasis |
| gldm_DependenceVariance | | gldm_LowGrayLevelEmphasis |
| gldm_GrayLevelNonUniformity | | gldm_SmallDependenceEmphasis |
| gldm_GrayLevelVariance | | gldm_SmallDependenceHighGrayLevelEmphasis |
| gldm_HighGrayLevelEmphasis | | gldm_SmallDependenceLowGrayLevelEmphasis |
| NGTDM features | | |
| ngtdm_Busyness | | ngtdm_Contrast |
| ngtdm_Coarseness | | ngtdm_Strength |
| ngtdm_Complexity | |  |
